# Diet and cardiovascular health in asymptomatic normo- and mildly-to-moderately hypercholesterolemic participants – baseline data from the BLOOD FLOW intervention study

**DOI:** 10.1186/1743-7075-10-62

**Published:** 2013-10-07

**Authors:** Maarit Hallikainen, Janne Halonen, Jussi Konttinen, Harri Lindholm, Piia Simonen, Markku J Nissinen, Helena Gylling

**Affiliations:** 1 Department of Clinical Nutrition, Institute of Public Health and Clinical Nutrition, University of Eastern Finland, P.O. Box 1627, 70211 Kuopio, Finland; 2 Finnish Institute of Occupational Health, Helsinki, Finland; 3 Heart and Lung Center, Helsinki University Central Hospital, Helsinki, Finland; 4 Department of Medicine, Divisions of Gastroenterology and Internal Medicine, University of Helsinki, Helsinki, Finland

**Keywords:** Diet, Saturated fatty acids, LDL cholesterol, Arterial stiffness, Endothelial function, Cardio-ankle vascular index, Reactive hyperemia index, Systematic cardiovascular risk estimation

## Abstract

**Background:**

For decades in Finland, intensive population strategies and preventive activities have been used to lower the risk of atherosclerotic coronary heart disease (CHD). Lifestyle changes, with the emphasis on diet, play an important role in preventive strategies. The aim of this study was to evaluate arterial stiffness and endothelial function in asymptomatic free-living adults and to relate the results to CHD risk factors and lifestyle habits with the emphasis on diet.

**Methods:**

Ninety-four asymptomatic participants were recruited by advertisements in four large companies and two research institutes employing mainly office workers. Arterial stiffness was assessed as the cardio-ankle vascular index in large arteries, and endothelial function as the reactive hyperemia index with peripheral arterial tonometry. The systematic Cardiovascular Risk Estimation (SCORE) was calculated.

**Results:**

The data was collected in the spring of 2011. Anthropometric, dietary, and lipid data was available from 92 participants, blood pressure from 85 and vascular measurements from 86–88 subjects (38% males; 62% females; mean age of all 51). The majority (72%) had an elevated low density lipoprotein (LDL) cholesterol concentration and over half were overweight or obese. SCORE stated that 49% of the participants had a moderate risk of cardiovascular disease. When compared to general recommendations, half of the participants had too high intake of total fat and in 66% the consumption of saturated fat was too high. In contrast, the intake of carbohydrates was too low in 90% of the participants and for fiber 73% were below recommendations. There was evidence of borderline or increased arterial stiffness in 72% of the participants and endothelial function was impaired in 8%. Arterial stiffness was associated with LDL cholesterol concentration (p = 0.024), dietary cholesterol intake (p = 0.029), and SCORE (p < 0.001).

**Conclusions:**

In a cross-sectional study of asymptomatic middle-aged participants, the half had a moderate risk for cardiovascular diseases manifested as increased arterial stiffness, elevated LDL cholesterol concentration, and poor dietary habits. The new observation that arterial stiffness was associated with dietary cholesterol intake and SCORE emphasizes the urgency of adequate lifestyle and dietary interventions to prevent future coronary events even in asymptomatic participants.

**Trial registration:**

Clinical Trials Register # NCT01315964

## Background

An elevated concentration of low-density lipoprotein (LDL) cholesterol is a major risk factor for atherosclerotic coronary heart disease (CHD) [1]. When the LDL cholesterol concentration is lowered by any means, then the risk for CHD is reduced [1]. Artery health can be evaluated non-invasively via surrogate markers i.e. arterial stiffness [2-5] and endothelial function [6]. The main beneficial lifestyle habits influencing the LDL cholesterol concentration are 1) low intake of cholesterol and total fats, especially those of saturated fats, 2) maintaining normal body weight, 3) ensuring regular physical activity [7]. It has been estimated that lowering the intake of saturated fat (<7E%) and cholesterol (<200 mg/d) can reduce the serum cholesterol concentration by about 5% [1]. The metabolic background of these effects has well been characterized.

In Finland in the late 1960s, CHD mortality was spectacularly high, especially in middle-aged men. Intensive population strategies and preventive activities to promote changes in lifestyle habits were initiated and gradually implemented throughout the entire country. The outcome has been monitored systematically in large population cohorts since 1972 by the Finnish National Institute for Health and Welfare [8]. The mortality rates declined in the whole population, especially in men by up to 80% from 1972 until about 2007. About 60% of this decline has been attributed to a reduction in the risk factors (serum cholesterol concentration, blood pressure and smoking). The lowered serum cholesterol concentration alone explained 45% of the decline in CHD mortality. Furthermore, the reduction in dietary fat and cholesterol explained 60-65% of the change in serum cholesterol concentration [9]. Reduced dietary saturated fat intake explained over 40% and that of dietary cholesterol about 9% of the serum cholesterol reduction. In contrast, the impact of cholesterol lowering medication was only 7-16%. In summary, dietary changes played a significant role in CHD risk reduction. Since the preventive aspects had succeeded over the long-time at the population level, the aim of the present study was to evaluate in asymptomatic free-living adults the indicators of vascular health and CHD risk factors. Furthermore, we evaluated how well the lifestyle recommendations, with an emphasis on diet, were followed in these participants in relation to the LDL cholesterol concentration and arterial stiffness and endothelial function.

## Methods

### Subjects

Ninety-four volunteers were screened and recruited into this 6-month intervention called BLOOD FLOW in 2011 by advertisements in the walls or in the intranet of four large companies employing mainly office workers and in two research institutes. This study is presenting the baseline data of the randomized, controlled, double-blind intervention evaluating the effects of plant stanol ester on cardiovascular health. The results of the intervention, which was carried out in 2011, have been published recently [10]. No inclusion or exclusion criteria for serum and lipoprotein lipids were set, but lipid-lowering medication or the consumption of nutrient supplements interfering with serum cholesterol concentration (red rice or berberine) were included as exclusion criteria. If the participants had used plant sterol/stanol products, they could be included in the study after a 3 weeks’ wash out period, which is enough for the serum cholesterol concentration to be returned to its initial value [11,12]. Other exclusion criteria were current gravidity or breast feeding, unstable coronary artery disease or coronary bypass or angioplasty <6 months, inflammatory bowel disease, alcohol consumption >45 g absolute alcohol/d, or abnormal liver, kidney or thyroid function. Possible medication should have remained unchanged for one month before the study. At recruitment, the participants contacted the research personnel, and their eligibility to the study was checked using a list of the exclusion criteria. All participants screened were invited to participate in the study. All data presented were collected from March to May 2011 after randomization.

The study was performed according to the principles of the Declaration of Helsinki. The Ethics Committee of the Department of Medicine, Hospital District of Helsinki and Uusimaa approved the study protocol. All participants gave their written informed consent.

### Measurements and laboratory analyses

Before blood samples were drawn body weight was measured with a digital scale and height with a stadiometer without shoes and in light clothes. Body mass index (BMI) was calculated as weight (kg) divided by height squared (m^2^). Waist circumference was measured at a level midway between the lowest rib and the iliac crest by the measuring tape.

Fasting blood samples were drawn after a 12 hour-fast. Plasma glucose and serum high-sensitive C-reactive protein (hs-CRP) concentrations were analyzed with routine standardized methods at the Central Laboratory of Helsinki University Hospital (HUSLAB). Serum total, LDL and high-density lipoprotein (HDL) cholesterol and serum triglycerides were analyzed enzymatically using automated analyzer systems. The level of non-HDL cholesterol was calculated as follows: non-HDL cholesterol = total cholesterol-HDL cholesterol.

### Vascular measurements

After 10 minutes’ supine rest, blood pressure was measured manually (Boso, Germany). The cardio-ankle vascular index (CAVI) was measured by the analysis of aortic pulse wave velocity (PWV) and pulse waveform (Vasera VS-1500, Fukuda Denshi Co, Japan) by the methods described elsewhere [4].

In short, PWV is obtained by dividing vascular length by the time taken for the pulse wave to propagate from the aortic valve to the ankle. CAVI is an index of arterial stiffness representing the elastic properties of arterial wall between aortic arch and distal arteries of the lower extremities and is considered to be independent of blood pressure at the time of measurement [2-5]. CAVI has proven to be valid and reproducible [13,14]. In large Japanese populations, CAVI ≥8 has been found to be an index of increased arterial stiffness, and if CAVI ≥9, then arterial stiffness can be considered as being significantly increased [15]. However, European reference values have not yet been published. CAVI has been proposed as being a surrogate marker for athero- or arteriosclerosis [4] and it has also been used as an indicator of vascular health during dietary modification [16].

Endothelial function was assessed with peripheral arterial tonometry (PAT) (Endo-PAT2000, Software version 3.0.3, Itamar Medical Ltd, Caesarea, Israel). The reactive hyperemia index (RHI) assesses the peripheral flow-induced arterial dilation after a provocation of reactive hyperemia, and it is defined as the ratio of the post-deflation pulse amplitude to the baseline pulse amplitude. Low values of RHI (≤1.67) reflect endothelial dysfunction [6]. The principal of PAT measurement has been described elsewhere [17]. The PAT measurement displays good reproducibility [18].

### Background questionnaire and nutrient intakes

Previous and present diseases, current drug treatment, use of vitamins or other nutrient supplements and lifestyle habits i.e. smoking habits, frequencies of alcohol consumption as well as frequencies of fitness or functional exercise were reviewed with a structured questionnaire.

All participants kept the 3-day food record between March and May 2011. One of the consecutive recording days was a weekend day. The portion sizes were estimated using household measures. The dietician contacted the participants by telephone and checked the amounts and qualities of foods in the hand-filled food records to clarify items that were unclear or missing. The same person coded all food records. The nutrients in food record were calculated by using the Diet32 dietary analysis program-version 1.4.6.2 (Aivo Ltd., Turku, Finland) which uses the Fineli® Food Composition Database (National Institute for Health and Welfare, Nutrition Unit, Helsinki, Finland). The ratio of calculated energy intake to estimated energy expenditure was 0.80 ± 0.20 indicating lower energy intake than the predicted one based on Schofield equations and cut-off factor (1.49) [19].

### Systematic cardiovascular risk estimation

The Systematic Cardiovascular Risk Estimation (SCORE), which estimates 10-year risk of fatal cardiovascular disease (CVD), was calculated by using the score charts with low risk based on following risk factors: age, sex, smoking, systolic blood pressure, and total cholesterol [20]. In addition, HDL cholesterol was taken into account in risk evaluation [21]. We defined “low risk” per SCORE guidelines as <1, “moderate risk” as 1–4 and “high risk” as 5–9.

### Statistical analyses

Statistical analyses were performed with SPSS for Windows 19.0 statistics program (SPSS, Chicago, IL, USA). Normality and homogeneity of variance assumptions were checked before further analyses. Logarithmic transformation was performed for variables that were not normally distributed or homogenous in variance.

In a large Japanese study, men had significantly greater CAVI than women in all age classes except for 70–74 years [22], therefore we assessed whether there were significant differences in vascular measurements between gender. For congruence we also assessed whether there were gender-related differences in serum lipids and lipoproteins, nutrient intake or other characteristics (Table 1).

**Table 1:** Characteristics of the participants and nutrient intakes

	**All (n = 92)**	**Men (n = 35)**	**Women (n = 57)**
Age (y)	50.8 ± 1.0	49.0 ± 1.7	51.8 ± 1.2
BMI (kg/m^2^)	25.2 ± 0.4	26.3 ± 0.5	24.5 ± 0.5^a^
Waist circumference (cm)	88.8 ± 1.1	94.9 ± 1.6	85.0 ± 1.2^a^
Systolic blood pressure (mmHg)	122 ± 1	124 ± 2	121 ± 2
Diastolic blood pressure (mmHg)	76 ± 1	77 ± 1	75 ± 1
Blood glucose (mmol/l)	4.92 ± 0.06	5.06 ± 0.09	4.83 ± 0.07^(a)^
hs-CRP (mmol/l)	1.05 ± 0.09	0.71 ± 0.09	1.25 ± 0.13^a^
Total cholesterol (mmol/l)	5.53 ± 0.09	5.44 ± 0.14	5.58 ± 0.12
LDL cholesterol (mmol/l)	3.53 ± 0.09	3.65 ± 0.14	3.45 ± 0.12
HDL cholesterol (mmol/l)	1.78 ± 0.05	1.57 ± 0.07	1.90 ± 0.06^a^
Triglycerides (mmol/l)	0.93 ± 0.04	1.01 ± 0.08	0.88 ± 0.05
non-HDL cholesterol (mmol/l)	3.75 ± 0.10	3.87 ± 0.16	3.67 ± 0.13
CAVI	8.66 ± 0.11	8.46 ± 0.18	8.78 ± 0.14
RHI	2.22 ± 0.06	2.16 ± 0.09	2.27 ± 0.08
Energy (MJ/d)	7.8 ± 0.2	8.4 ± 0.4	7.5 ± 0.2
Protein (% of energy)	17.3 ± 0.3	18.4 ± 0.6	16.7 ± 0.4^a^
Fat (% of energy)	34.1 ± 0.7	32.9 ± 1.2	34.8 ± 0.8
SFA (% of energy)	11.4 ± 0.3	11.5 ± 0.6	11.3 ± 0.4
MUFA (% of energy)	11.8 ± 0.3	11.3 ± 0.5	12.1 ± 0.4
PUFA (% of energy)	5.6 ± 0.2	5.1 ± 0.3	5.9 ± 0.3^a^
Carbohydrates (% of energy)	41.5 ± 0.7	40.5 ± 1.3	42.2 ± 0.9
Alcohol (% of energy)	2.4 ± 0.3	3.7 ± 0.7	1.6 ± 0.3^a^
Cholesterol (mg/d)	222.6 ± 10.1	247 ± 18.2	207.7 ± 11.5^a^
Cholesterol (mg/MJ)	29.1 ± 1.4	30.1 ± 2.0	28.5 ± 1.8
Fiber (g/d)	21.3 ± 0.7	20.3 ± 1.2	21.8 ± 0.9
Fiber (g/MJ)	2.8 ± 0.1	2.5 ± 0.1	2.9 ± 0.1^a^

Values shown are means ± SEM. BMI = body mass index; CAVI = cardio-ankle vascular index; HDL-C: high density lipoprotein; hs-CRP = high sensitive C-reactive protein; LDL: low density lipoprotein; MUFA: monounsaturated fatty acid; PUFA: polyunsaturated fatty acid; RHI: reactive hyperemia index; SFA: saturated fatty acid.

n = 33/52 (men/women) for systolic and diastolic blood pressure, n = 34/54 for CAVI and n = 34/52 for RHI.

^a^p < 0.05 or less significantly different from men as analyzed by Student’s t-test or Mann–Whitney U-test (alcohol).

^(a)^p > 0.05 after adjustment with BMI, waist circumference, intakes of protein, PUFA, alcohol, cholesterol (mg/d) and fiber (g/MJ) as analyzed by analysis of covariance.

Student’s t test was used to assess differences between gender and univariate analysis of variance was used to assess differences between the grades of artery stiffness for continuous variables. Mann–Whitney U-test was used in the gender-comparison and Kruskal-Wallis test was used in the comparison of the grades of arterial stiffness for a non-normally distributed variable (alcohol intake). The differences between gender were further assessed using analysis of covariance (ANCOVA) including variables that significantly differed between gender as covariates (see Table 1). Similarly, the differences between the grades of arterial stiffness were assessed using ANCOVA including the covariates described in Table 2.

**Table 2:** Diseases, medication and smoking in the participants of the study

	**All**	**Men**	**Women**
	**(n = 92)**	**(n = 35)**	**(n = 57)**
**Diseases**^a^			
Hypertension (n)	15	4	11
Diabetes or impaired glucose tolerance (n)	3	1	2
Cancer, remission (n)	6	3	3
Cholelithiasis (n)	2	0	2
Arthritis (n)	1	0	1
Celiac disease (n)	2	0	2
Hypothyreosis (n)	5	0	5
Asthma (n)	5	1	4
**Hypertension**^a^			
Calcium channel blockers (n)	3	1	2
Beta blockers (n)	2	0	2
Diuretics (n)	2	2	0
Angiotensin converting enzyme- or angiotensin receptor blocking agents (n)	7	3	4
**Hormonal medication**^a^			
Thyroxin (n)	6	0	6
Contraceptives (n)	4	-	4
Hormone replacement therapy (n)	15	-	15
**Smoking** (n)^a^	7	5	2

^a^No significant differences were found between the gender as analyzed by Fisher exact test.

Relationships between LDL cholesterol, hs-CRP, CAVI and RHI as well as relationships between CAVI and RHI and nutrient intakes were analyzed by Pearson correlation test. In addition, the relationship between SCORE and above variables was analyzed by the Spearman correlation test. In addition, to evaluate the effects of gender and age on associations of serum LDL cholesterol and CAVI, a multiple linear regression analysis was used. Fisher exact test was used for categorical measures. The Bonferroni adjustment was used to control the overall α level in multiple comparisons. A p-value of <0.05 was considered statistically significant. The results are given as means ± SEM, unless otherwise indicated.

## Results

### Characteristics

Baseline data was available from 92 participants except for blood pressure n = 85 and vascular measurements n = 86-88 (Table 1) due to technical problems. The majority of the participants (62%) were women. All participants were aged between 24–66 years. The prevalence of diseases, medication and, smoking habits are given in Table 2. None of the participants had CHD or other cardiovascular diseases. Of the fifteen participants with hypertension, nine were taking regular medication. All participants with a history of hypothyreosis were receiving thyroxin therapy and were euthyreoid. Type 2 diabetes (n = 1) was controlled by diet alone. The other diseases were under good control or in remission. According to the questionnaire, 25% of the participants took physical exercise four times or more weekly, 44% two to three times weekly, 23% once a week and 9% stated that they were physically inactive.

BMI, waist circumference and plasma glucose concentration were higher and the serum hs-CRP and HDL cholesterol concentrations lower in men than in women. There were no significant gender differences in the other clinical variables or frequencies of diseases or medications. The intakes of protein, alcohol and cholesterol (mg/d) were higher and the intakes of polyunsaturated fatty acids (PUFA) and fiber (g/d) lower in men than in women, but there were no other differences in nutrient intakes between the genders. The gender difference in serum hs-CRP and HDL cholesterol concentrations remained significant after adjustment with BMI, waist circumference and the above-mentioned nutrient intakes i.e. protein, PUFA, alcohol, cholesterol and fiber.

### Characteristics of the participants vs. reference values

**Figure 1:**
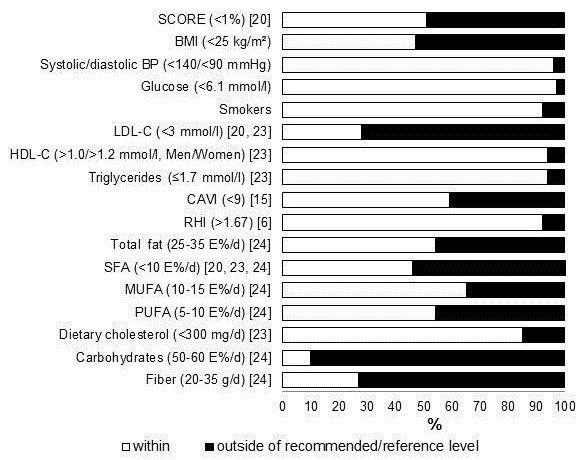
**Distribution of participants within or outside of recommended/reference value of different variables.** BMI: body mass index; BP: blood pressure; CAVI: cardio-ankle vascular index; HDL-C: high density lipoprotein cholesterol; E%: percent of energy; LDL-C: low density lipoprotein cholesterol; MUFA: monounsaturated fatty acid; PUFA: polyunsaturated fatty acid; RHI: reactive hyperemia index; SCORE: Systematic Cardiovascular Risk Estimation; SFA: saturated fatty acid.

BMI ranged from 17.9 to 36.6 kg/m^2^. About 47% of the whole study population had normal weight (BMI < 25 kg/m^2^) and 53% were overweight or obese (BMI 25–37 kg/m^2^) (Figure 1). The majority of the men (77%) were overweight or obese as compared with the women (39%) (χ^2^ = 12.943, p < 0.001). About one in four (26%) of all participants had abdominal obesity (waist circumference >102 cm in men and >88 cm in women).

Serum total and LDL cholesterol varied from 3.4 to 7.2 mmol/l and from 1.5 to 5.6 mmol/l. The mean values are given in Table 1. Sixty-six participants had elevated serum total (≥ 5.0 mmol/l [20,23]) and LDL cholesterol (≥3.0 mmol/l [20,23]) concentrations (Figure 1). The HDL cholesterol concentration was low in four men (≤1.0 mmol/l [23]) and in two women (≤1.2 mmol/l [23]) and that of triglycerides high (>1.7 mmol/l [23]) in six participants. The magnitudes of the frequencies were similar in men and women.

SCORE was in 51% of the participants below 1% (low risk) whereas in 49% of the participants, the risk was 1-4% (moderate risk). In addition, 35 participants (84%) belonging to low risk class had LDL cholesterol ≥2.5 mmol/l, which requires to initiate lifestyle changes even in this risk class [20]. None of the participants belonged to the high risk group.

About 28% of the participants had normal arterial stiffness (CAVI < 8), 31% had slightly elevated arterial stiffness (CAVI 8–9) and 41% had elevated arterial stiffness (CAVI >9 [15]) (Figure 1). Seven participants had abnormal endothelial function (RHI ≤ 1.67 [6]) (Figure 1). No gender differences were found in the frequencies in CAVI or RHI groups.

### Dietary intakes vs. dietary recommendations

According to the current dietary recommendations, the total fat intake was too high (25–35 percent of energy (E%) [24]) in 46% of the participants, that of saturated fatty acid (SFA) intake (<10E% [20,23,24]) in 66% of the participants, and dietary cholesterol intake (<300 mg/d [23] in 15% of the participants (Table 1 and Figure 1). The dietary recommendation for monounsaturated fatty acid (MUFA) intake (10-15E% [24]) was not being met by 35% and that of PUFA intake (5-10E% [24]) by 46% participants. The carbohydrate intake was less than recommended (50-60E% [24]) in 90% of the participants and in 39% it was less than 40E%. The recommendation of fiber intake (25–35 g/d [24]) was not fulfilled in 73% of the participants. No gender differences were found in nutrient intakes vs. dietary recommendations.

### Arterial stiffness

When participants were divided according to their arterial stiffness (Table 3), it was noted that age and systolic blood pressure were lower in the participants with CAVI < 8 and CAVI 8–9 and the diastolic blood pressure was lower in the participants with CAVI 8–9 as compared with the participants with CAVI > 9. In addition, energy intake was greater, but cholesterol (mg/MJ) intake lower in those participants with CAVI < 8 compared with the participants with CAVI > 9. After adjustment for gender, age and the intakes of energy and cholesterol (mg/MJ), it was found that systolic blood pressure was still lower in the participants with CAVI 8–9 as compared with the participants with CAVI > 9.

**Table 3:** Characteristics of the participants and nutrient intakes related to arterial stiffness

	**CAVI < 8 (n = 25)**	**CAVI 8–9 (n = 27)**	**CAVI > 9 (n = 36)**
Men/women (n)	11/14	12/15	11/25
Age (y)	43.6 ± 1.9	48.2 ± 1.5	57.1 ± 1.0^a,b^
BMI (kg/m^2^)	24.8 ± 0.7	25.3 ± 0.8	25.3 ± 0.5
Waist circumference (cm)	86.9 ± 1.8	90.4 ± 2.4	89.0 ± 1.5
Systolic blood pressure (mmHg)	120 ± 2	117 ± 2	128 ± 2^(a),b^
Diastolic blood pressure (mmHg)	75 ± 1	73 ± 1	79 ± 1^(b)^
Blood glucose (mmol/l)	4.75 ± 0.09	4.96 ± 0.12	5.00 ± 0.09
hs-CRP (mmol/l)	0.74 ± 0.13	1.09 ± 0.17	1.14 ± 0.16
Total cholesterol (mmol/l)	5.24 ± 0.20	5.44 ± 0.15	5.72 ± 0.14
LDL cholesterol (mmol/l)	3.26 ± 0.16	3.52 ± 0.16	3.70 ± 0.16
HDL cholesterol (mmol/l)	1.79 ± 0.09	1.71 ± 0.08	1.76 ± 0.08
Triglycerides (mmol/l)	0.77 ± 0.07	0.99 ± 0.10	1.00 ± 0.07
non-HDL cholesterol (mmol/l)	3.45 ± 0.17	3.73 ± 0.17	3.96 ± 0.17
CAVI	7.34 ± 0.11	8.60 ± 0.05^a^	9.62 ± 0.09^a,b^
RHI	2.15 ± 0.13	2.10 ± 0.08	2.38 ± 0.09
Energy (MJ/d)	8.7 ± 0.4	7.9 ± 0.4	7.2 ± 0.3^a^
Protein (% of energy)	17.6 ± 0.8	17.5 ± 0.7	17.2 ± 0.5
Fat (% of energy)	33.2 ± 1.1	33.7 ± 1.4	35.1 ± 1.1
SFA (% of energy)	11.6 ± 0.6	10.9 ± 0.5	11.7 ± 0.6
MUFA (% of energy)	11.0 ± 0.5	11.7 ± 0.8	12.4 ± 0.5
PUFA (% of energy)	5.4 ± 0.3	5.6 ± 0.4	5.7 ± 0.3
Carbohydrates (% of energy)	42.2 ± 1.2	41.8 ± 1.5	40.9 ± 1.1
Alcohol (% of energy)	2.2 ± 0.8	2.4 ± 0.7	2.2 ± 0.5
Cholesterol (mg/d)	209.0 ± 16.9	214.3 ± 20.5	234.6 ± 16.6
Cholesterol (mg/MJ)	23.9 ± 1.4	26.8 ± 2.1	33.7 ± 2.7^a^
Fiber (g/d)	22.3 ± 1.4	21.7 ± 1.3	20.3 ± 1.1
Fiber (g/MJ)	2.6 ± 0.1	2.8 ± 0.2	2.9 ± 0.1

Values shown are means ± SEM. BMI = body mass index; CAVI = cardio-ankle vascular index; HDL-C: high density lipoprotein; hs-CRP = high sensitive C-reactive protein; LDL: low density lipoprotein; MUFA: monounsaturated fatty acid; PUFA: polyunsaturated fatty acid; RHI: reactive hyperemia index; SFA: saturated fatty acid

CAVI < 8 depicts normal arterial stiffness; CAVI 8–9 depicts slightly elevated arterial stiffness; CAVI > 9 depicts markedly elevated arterial stiffness.

n = 24/27/34 (CAVI < 8/8-9/>9) for systolic and diastolic blood pressure, n = 25/27/36 for CAVI and n = 25/27/34 for RHI.

^a^p < 0.05 from the participants with CAVI < 8 as analyzed by univariate analysis of variance with Bonferroni correction. ^(a)^p > 0.05 after adjustment for age, gender and the intakes of energy and cholesterol (mg/MJ) systolic blood pressure did not differ from the participants with CAVI < 8.

^b^p < 0.05 from the participants with CAVI 8–9 as analyzed by univariate analysis of variance with Bonferroni correction. ^(b)^p > 0.05 after adjustment for age, gender and the intakes of energy and cholesterol (mg/MJ) diastolic blood pressure did not differ from the participants with CAVI 8–9.

### Associations

LDL cholesterol and CAVI were positively associated with age (r = 0.244, p = 0.019 and r = 0.667, p < 0.001, respectively) and CAVI tended to correlate with hs-CRP (r = 0.205, p = 0.055). The LDL cholesterol concentration was associated with CAVI (r = 0.240, p = 0.024), especially in the participants with the elevated LDL cholesterol ≥3 mmol/l [20,23] (r = 0.305, p = 0.030 vs. LDL cholesterol <3 mmol/l r = 0.169, p = 0.838). However, these associations did not remain significant after adjustment for gender and age. LDL cholesterol and RHI were not correlated with each other. CAVI was positively associated with dietary cholesterol (mg/MJ/d) intake (r = 0.233 p = 0.029) even after adjustment for gender and age (β = 0.200, p = 0.016). CAVI was not associated with any other nutrient intake. RHI was neither associated with nutrient intakes nor with CAVI.

SCORE was positively correlated with LDL cholesterol (r = 0.251, p = 0.020) and CAVI (r = 0.368, p < 0.001). SCORE was not correlated with RHI (r = 0.054, p = 0.621).

## Discussion

The novel observations in the present study were that the majority (72%) of asymptomatic middle-aged participants had elevated LDL cholesterol concentration, over half were overweight or obese, and 72% displayed borderline or increased arterial stiffness. As assessed with the SCORE [20,21], only half of the participants could be categorized into the low risk group, and furthermore when the LDL cholesterol concentration was taken into consideration, then only seven individuals could be considered as real low-risk persons who would not benefit from lifestyle intervention. The SCORE was associated with arterial stiffness. Regarding the risk patterns of this study population, it can be stated that their dietary habits were far from ideal. Over two thirds were consuming too much saturated fat, 90% consumed too little carbohydrates, and about 70% too little fiber. Of the other lifestyle habits, although only 8% of the participants were current smokers, for the vast majority physical activity did not reach the recommended levels [24].

These results suggest that a large number of the asymptomatic participants had increased CHD risk factors and increased arterial stiffness. This finding may reflect the same phenomenon observed in a recent survey conducted by the Finnish National Institute for Health and Welfare, which demonstrated that in 2012, the serum cholesterol concentrations have increased from those values observed in 2007. This represents the end of what has been a beneficial trend which started decades ago [8,25]. The recent European guidelines on cardiovascular disease prevention in clinical practice emphasize that a healthy diet is the cornerstone of cardiovascular disease prevention e.g. SFA intake should not be more than 10% of total energy intake [20,26]. Furthermore, according to European guidelines, functional foods containing phytosterols (plant sterols and stanols) can effectively lower LDL cholesterol levels by on average 10% when consumed in amounts of 2 g/day [20]. This cholesterol lowering effect is additional to that obtained with a low saturated fat diet or the use of statins. With respect to the cardiovascular risk level (SCORE) in the present study, it would have been high enough to recommend the initiation of the intervention strategies with dietary changes in almost all of the participants [20,26]. Recent research has revealed that even individuals with a low risk of vascular events benefit considerably from intensive LDL cholesterol lowering [27].

Arterial health can be assessed with surrogate markers evaluating arterial stiffness and endothelial function. Arterial stiffness expressed as PWV and endothelial function measured as pulse wave amplitude during reactive hyperemia are novel, non-invasive methods with which to assess subclinical atherosclerosis, even to predict future cardiovascular events [28,29]. The measurement of endothelial function by PAT has recently been used in nutrition-based interventions [30,31]. Arterial stiffness and endothelial function evaluate different aspects of the arterial wall and need not correlate with each other. In the present study, RHI was not impaired in this population and this parameter did not correlate with LDL cholesterol concentration, SCORE or with any of the dietary variables. These results indicate that if one wishes to undertake a comprehensive evaluation of vascular health the surrogate markers both of endothelial function and arterial stiffness are needed.

CAVI is an index of arterial stiffness in large arteries reflecting the elastic properties of the arterial wall between the aortic arch and the lower extremities [2-4]. CAVI increases with age and in arteriosclerotic diseases, and is related to the serum LDL cholesterol concentration and to elevated blood pressure [4]. CAVI was elevated in every fifth male Finnish firefighter (mean age 48 yrs) [3]. Aerobic fitness in addition to age was correlated with CAVI [3]. In the present study, which evaluated a slightly older population of mainly white-collar employees, the frequency of impaired CAVI was much greater. In addition to age and the LDL cholesterol concentration, the value of CAVI correlated with the SCORE. This finding suggests that interventions should focus especially on those risk factors which can be modified. In this study population with rather well-controlled smoking and blood pressure, a reduction in the LDL cholesterol concentration would be of special importance. The observation that dietary cholesterol intake was associated with CAVI is important because it connects the dietary habits to arterial well-being.

There are some limitations in this study. There were four participants who had used plant sterol/stanol products before the study, but they were included in the study after a 3 weeks’ wash out period, which is enough for the serum cholesterol concentration to be restored to its initial value [11,12]. Excluding these four individuals from all statistical analyses did not change the results. In the cross-sectional study, data was collected only at one time point, and thus it is difficult to prove causalities for the associations found. In addition, the study population was of limited size, they volunteered to take part in the intervention, and therefore one cannot extrapolate these results to the general population. However, the study population represented a homogenous population of urban, well-educated, middle-aged participants interested in their health and well-being. Food recording for three to four consecutive days has been found to be sufficient to estimate the intake of energy nutrients at a group level [32,33]. However, for the monitoring of cholesterol intake, more recording days may be needed [32]. In general, energy intake as measured by dietary records has been found to be 10-30% lower as compared with total energy expenditure measured by doubly-labeled water [34]. Because of the under-reporting, all the intakes have been energy-adjusted, either as percentages of energy or per MJ except for the cholesterol intake was also expressed as mg/d and fiber intake as g/d.

## Conclusions

In a cross-sectional study the majority of asymptomatic, middle-aged participants had an elevated LDL cholesterol concentration, increased arterial stiffness and poor dietary habits with high intake of SFA. The novel observation that arterial stiffness was associated with dietary cholesterol intake and SCORE emphasizes the urgency of adequate lifestyle and dietary interventions to prevent future coronary events.

## Abbreviations

BMI: Body mass index; BP: Blood pressure; CAVI: Cardio-ankle vascular index; CHD: Coronary heart disease; CVD: Coronary vascular disease; HDL: High density lipoprotein; hs-CRP: High sensitive C-reactive protein; E%: Percent of energy; LDL: Low density lipoprotein; MUFA: Monounsaturated fatty acid; PAT: Peripheral arterial tonometry; PUFA: Polyunsaturated fatty acid; PWV: Aortic pulse wave velocity; RHI: Reactive hyperemia index; SCORE: Systematic cardiovascular risk estimation; SFA: Saturated fatty acid.

## Competing interests

The authors declare that they have no competing interests.

## Authors’ contributions

HG, HL, MH and MN were responsible for the study design. HG, HL, MH, JH, JK, PS and MN carried out the study. PS was responsible for the collection of all data except the nutrient data, which was collected by MH, who also performed the statistical analysis. All authors participated in drafting the manuscript. All authors read and approved the final manuscript.

## Authors’ information

This study is dedicated to the memory of late Professor Tatu A. Miettinen.
